# Supplementary material for: Malnutrition-Related Health Outcomes in Older Adults with Hip Fractures: A Systematic Review and Meta-Analysis
Source: Nutrients. 2024 Apr 5;16(7):1069. doi: 10.3390/nu16071069 (PMC11013126; doi:10.3390/nu16071069)
Supplement: Supplementary file 1 [file nutrients-16-01069-s001.zip › Supplementary Table S1. Search strategy.pdf]

Supplementary Table S1. Search strategy.

**Database**	**Search details**
**PubMed**	("Nutritional Status"[Mesh] OR MNA[Title/Abstract] OR CONUT[Title/Abstract] OR PNI[Title/Abstract] OR GNRI[Title/Abstract])<br>AND (risk)<br>AND (outcome)<br>AND (((hip[Title/Abstract] OR femoral[Title/Abstract]) AND (injury[Title/Abstract] OR fracture[Title/Abstract]))<br>OR "Proximal Femoral Fractures"[Mesh] OR "Femoral Neck Fractures"[Mesh] OR "Hip Fractures"[Mesh])
**Web of Science**	Topic:"Nutritional Status" (Mesh) OR MNA OR CONUT OR PNI OR GNRI<br>AND risk<br>AND outcome<br>AND<br>Topic: (("Proximal Femoral Fractures"[Mesh] OR "Femoral Neck Fractures"[Mesh] OR "Hip Fractures"[Mesh]) OR (hip OR femoral) AND (injury OR fracture))
**Scopus**	TAK: ("Nutritional Status" [mesh] OR mna OR conut OR pni OR gnri)<br>AND risk<br>AND outcome<br>AND<br>TAK: (("Proximal Femoral Fractures"[Mesh] OR "Femoral Neck Fractures"[Mesh] OR "Hip Fractures"[Mesh]) OR (hip OR femoral) AND (injury OR fracture))
