# Supplementary material for: Malnutrition-Related Health Outcomes in Older Adults with Hip Fractures: A Systematic Review and Meta-Analysis
Source: Nutrients. 2024 Apr 5;16(7):1069. doi: 10.3390/nu16071069 (PMC11013126; doi:10.3390/nu16071069)
Supplement: Supplementary file 1 [file nutrients-16-01069-s001.zip › Supplementary Table S2. New Castle Ottawa Scale.pdf]

Supplementary Table S2A. New Castle Ottawa Scale of the studied selected of the Cohort studies.

Cohort Study	Representativeness of the exposed cohort	Selection of the unexpose d cohort	Ascertainme nt of exposure	Outcome of interest not present at start of study	Control for		Assessment of outcome	Follow-up long enough for outcomes to occur	Adequacy of follow-up of cohorts	Total quality score
					important factors	additional factors				
34	X	X	X	X	X	X		X	X	8
39	X	X	X	X	X	X	X	X	X	9
43	X	X	X	X	X	X	X		X	8
49	X	X	X	X	X	X	X		X	8
51		X	X	X		X	X	X	X	7
56	X	X	X	X	X		X	X	X	8
64	X	X	X	X			X	X	X	7
68	X	X	X	X	X	X	X		X	8
70	X	X	X	X		X	X	X	X	8
72	X	X	X	X	X	X	X	X	X	9

Supplementary Table S2B. New Castle Ottawa Scale of the studied selected of the Case-Control studies.

Case-Control Study	Adequate definition of cases	Representativenes s of cases	Selection of controls	Definition of controls	Comparability		Ascertainment exposure	Same method of ascertainment for cases and controls	Non-Response rate	Total quality score
					important factors	additional factors				
36	X	X	X	X	X		X	X	X	8
53	X	X	X	X	X	X	X	X	X	9
67	X	X	X	X			X	X	X	7
69	X	X	X	X			X	X	X	7
